# Pharmacists reinventing their roles to effectively respond to COVID-19: a global report from the international pharmacists for anticoagulation care taskforce (iPACT)

**DOI:** 10.1186/s40545-020-00216-4

**Published:** 2020-06-17

**Authors:** Filipa Alves da Costa, Vivian Lee, Silvana Nair Leite, Maria Dolores Murillo, Tom Menge, Sotiris Antoniou

**Affiliations:** 1 Faculty of Pharmacy, University of Lisbon, Av. Prof. Gama Pinto, 1649-003 Lisbon, Portugal; 2 The Chinese University of Hong Kong, Central Ave, Shatin, Hong Kong; 3 Federal University of Santa Catarina, R. Eng. Agronômico Andrei Cristian Ferreira, s/n - Trindade, Florianópolis, SC 88040-900 Brazil; 4 Sociedad Española de Farmacia Familiar y Comunitaria (SEFAC), Paseo de las Delicias 31, 28045 Madrid, Spain; 5 Kenyatta National Hospital, Hospital Rd, Nairobi, Kenya; 6 Barts Health NHS Trust, W Smithfield, London, EC1A 7BE UK

## Abstract

SARS-CoV2 is dramatically impacting the global population. Worldwide, pharmacists are changing their roles and being increasingly recognized for their role as essential service providers. This commentary provides some examples collected from Asia, Europe, the Americas and Africa, ranging from essential services to meet human rights basic needs, extended generalist services developed to ensure continuity of care and supply of essential medicines to the development of differentiated extended responsibilities in emergency care. All examples were collected using a network of pharmacists from 27 countries, representing various areas of pharmacy practice, education and research and outreaching to include patient advocates. Selected services illustrate good practice, capability to adapt and contribution to universal health coverage. Above all, it demonstrates the commitment and innovation of the pharmaceutical workforce in the fight against COVID-19.

## Impact of COVID-19 on the pharmacists’ roles and image

As a result of an unprecedent pandemic, caused by SARS-CoV2, pharmacists worldwide are increasingly recognized for their role as providers of an essential service. This recognition partly results from their unique position to ensure the supply of essential medicines, but also because pharmacists are demonstrating to be a dynamic workforce, highly committed to the health of the population, and who competently deliver various services and products meeting current societal demands. Pharmacists are part of the healthcare team and work aside with policy makers to find solutions and overcome barriers. Organizations focused on pharmacists, such as the International Pharmaceutical Federation (FIP) issued guidance for pharmacy teams on COVID-19 [1], already adapted to tailor the needs of specific countries, including Pakistan [2]. Organizations beyond pharmacy are also issuing guidance targeted at pharmacists, including the Center for Disease Control and Prevention, clearly showing the scope of pharmacists’ interventions in fighting COVID-19 [3].

iPACT as an expert network of pharmacists from 27 countries, representing various areas of pharmacy practice, education and research, and include patient advocates, has selected some examples of pharmacists’ extended role in time of crisis, to illustrate good practice, capability to adapt and contribution to universal health coverage.

### Extended pharmacists’ services in Asia

Mainland China, where the outbreak was first identified, has two separate administrative regions: Hong Kong and Macau. In Hong Kong, there are a total of 1013 confirmed/probable COVID-19 cases and 4 deaths related to COVID-19 as of 15 April 2020. Due to the government newly implemented regulations on social distancing, citizens are not allowed to have any public gathering with four people or more. The hospital authority has issued special guidance recommending people who have been outside Hong Kong in the previous 14 days not to enter specialist outpatient clinics. This arrangement is in line with the compulsory quarantine guidelines on people entering Hong Kong from other countries and Mainland China. High risk individuals, including elders, chronically ill and immunosuppressed patients, are advised not to leave home during the current COVID-19 pandemic, leading to issues around medication supply. Thus, pharmacists developed alternative services to ensure continued treatment. In the community setting, a group involving community pharmacists working collaboratively with physicians initiated a new medication refill service, anticipating the possibility for patients to access their hospital medication through a community pharmacy, instead of having to visit the outpatient pharmacy in the public hospital. The community outreach team at the Chinese University of Hong Kong initiated the provision of care packages, including masks, alcohol-based hand sanitizers, toilet paper, and rice packages for community-dwelling elderly. This team’s extended services also comprise weekly telephone interviews as part of disease management to optimise medicines use and medication adherence.

### Extended pharmacists’ services in Europe

In many countries, including the United Kingdom, hospital pharmacists are engaging in tasks outside of their normal remit – developing and refreshing their skills in critical care and managing COVID-19 patients directly, supporting helplines and being an integral member of the national emergency team. For many pharmacists this meant taking additional responsibilities and assuming new risks, requiring changes in regulations and support provided by bodies such as the General Pharmaceutical Council (GPhC). Ethical decision-making frameworks were created to support pharmacists and pharmacy teams faced with difficult decisions; and additional insurance to cover for these new risks was anticipated. As a measure to expand the workforce, temporary registration of pharmacists that had left the register was granted to meet the increasing demands of the population. A joint statement was also signed by the GPhC and the Royal Pharmaceutical Society to permit using volunteers to support pharmacists in delivering medicines [4].

Spain is currently the second most affected country worldwide, with 34,620 affected individuals and 3500 deaths/100,000 inhabitants. Community pharmacists continue working despite the threat, to ensure medicines are available for all that need them. There are reports of over 450 pharmacists infected by COVID-19 by the 8th April [5], but that does not stop them from filling their duty and reinventing new services and ways of working. The Spanish society SEFAC has proposed to the government various innovative services, including risk assessment for COVID-19 and involvement in the distribution of self-tests. The main approved regulatory change is home delivery of medicines, to ensure continued supply of medicines for pre-existing conditions [6]. Community pharmacists’ role in raising public awareness of preventive measures and advising on symptomatic medications during isolation are visible every day. Remote education also developed through livestreaming initiatives directed at high-risk groups (eg. respiratory conditions) [7].

In Portugal, a new decree was just approved to enable community pharmacies to deliver hospital-only medicines for extended periods so that people are not deprived of essential medicines. Also, for people living with chronic illness, a decree was published enabling automatic renewal of prescriptions [8, 9]**.**

### Extended pharmacists’ services in the Americas

In Brazil, as part of the unique public system (known as SUS), pharmacists target their services to vulnerable groups of the population, including people living with HIV. In this area, remote solutions for the continued supply of high-cost medicines medications used in transplants, multiple myeloma, SARS prevention, amongst others, were immediately implemented. Services included telephone appointments provided by students and professors of the Pharmacy school verifying the supply and advising on responsible use of medicines. The regulatory agency issued a special program to allow continued supply of controlled substances, including benzodiazepines and antipsychotics, for longer periods [10]. This regulation establishes that the pharmacist is accountable for individual risk assessment prior to deciding on appropriateness of therapy and thus refilling prescriptions. While enabling supply of essential medicines, additional tasks were also developed to ensure the safety of professionals involved; as an example, pharmacists developed their own alcohol-based gels for hand sanitizing, which were in short supply. As a side measure, the public system has also agreed that community pharmacists would be leading the Influenza immunization campaign in 2020, so that primary care units are less overburden.

### Extended pharmacists’ services in Africa

In the global context, the African continent has comparatively low numbers of infections by COVID-19. There are reports suggesting heard immunity to COVID-19 created by malaria, possibly a result of being a blood infection unlike what has been believed so far. The connections between these two infections also refer to disruptions in supply chain of essential malaria preventive products, medications and healthcare services. This crisis highlighted that a strong investment is needed in African pharmaceutical industry to become self-sufficient as currently only a minor proportion of active ingredients are available. Community pharmacies in Kenya constitute a strong pillar of primary care offer, as the country strives to achieve universal health coverage. When many other essential services are providing only emergency care, frontline pharmacists keep doors open, maintaining services accessible to all who need it. A role adopted by pharmacists is combating misinformation, aggravated by media reports and by low literacy levels. In addition, running water and soap is also provided for people to handwashing while waiting.

### Beyond traditional pharmacists’ services

There are also side-effects of COVID-19 that mostly relate to the effect of control measures imposed, including complete lock downs. There are reports of increases in domestic violence, which have resulted in initiatives led by the civil society in association with pharmacists that consist of public campaigns advising victims to go to the nearest pharmacy and request a mask-19. Pharmacists faced with this request immediately report the situation. A measure currently active in Spain and France. In the UK, pharmacies have adapted pharmacy consultation rooms to safe places for victims of domestic violence.

## Conclusion

These are a few examples of extended services being provided globally, which clearly highlight the commitment and innovation of pharmaceutical workforce in the fight against COVID-19. More examples could be mentioned, including from Oceania, but we opted for highlighting the original, the current and possible future epicentrics of COVID-19.

## Data Availability

Not applicable.

## References

[1] International Pharmaceutical Federation. Coronavirus/COVID-19 preparedness. Available on: https://www.fip.org/coronavirus. Accessed 17 Apr 2020.

[2] Bukhari N, Rasheed H, Nayyer B, Babar Z (2020). Pharmacists at the frontline beating the COVID-19 pandemic. J Pharmaceut Policy Pract.

[3] Center for Disease Control and Prevention. Considerations for Pharmacies during the COVID-19 Pandemic. Available on: https://www.cdc.gov/coronavirus/2019-ncov/hcp/pharmacies.html. Accessed 19 Apr 2020.

[4] General Pharmaceutical Council. Latest updates on COVID-19 (coronavírus). Available on: https://www.pharmacyregulation.org/contact-us/coronavirus-latest-updates. Accessed 17 Apr 2020.

[5] General Council of Official Colleges of Pharmacists of Spain, COVID-19 updates. Published in Portalfarma, The number of infected professionals increases 66% and 65 pharmacies remain shut. “Aumenta un 66% el número de profesionales infectados y 65 farmacias permanecen cerradas” [available in Spanish], available on: https://www.portalfarma.com/Profesionales/consejoinforma/Paginas/2020-registro-abril-afectados-coronavirus-farmacias-comunitarias.aspx. Accessed 10 Apr 2020.

[6] Baixauli VJ, Satué E, Murillo Mª D, Molinero A, Gómez JC, Estrada G (2020). Propuesta para la dispensación y entrega de medicamentos y prodcutos sanitários en el domicílio del paciente desde la farmácia comunitária durante el estado de alarma por COVID-19. Madrid: Sociedad Española de Farmacia Familiar y Comunitária (SEFAC).

[7] Sociedad Española de Farmacia Familiar y Comunitária (SEFAC). Available on: https://www.sefac.org/sesion-online-covid-19-y-enfermedades-respiratorias-cronicas. Accessed 10 Apr 2020.

[8] Circular Normativa n° 005/CD/550.20.001. Issued 07/04/2020. Guidance on access to hospital only medication through community pharmacies in the context of COVID-19 pandemic [available in Portuguese]. Available on: https://www.infarmed.pt/web/infarmed/infarmed/-/journal_content/56/15786/3624277. Accessed 10 Apr 2020.

[9] Portaria n° 90-A/2020. Issued 09/04/2020. Exceptional and temporary measures for electronic prescribing of medicines during COVID-19 pandemic [available in Portuguese]. Available on: https://dre.pt/pesquisa/-/search/131338887/details/maximized. Accessed 10 Apr 2020.

[10] ANVISA warning 20280. Available on: http://www.in.gov.br/en/web/dou/-/resolucao-rdc-n-357-de-24-de-marco-de-2020-249501721. Accessed 10 April 2020.

